# Genetic Diversity of *Brucella* Reference and Non-reference Phages and Its Impact on *Brucella*-Typing

**DOI:** 10.3389/fmicb.2017.00408

**Published:** 2017-03-15

**Authors:** Jens A. Hammerl, Cornelia Göllner, Claudia Jäckel, Holger C. Scholz, Karsten Nöckler, Jochen Reetz, Sascha Al Dahouk, Stefan Hertwig

**Affiliations:** ^1^Department of Biological Safety, German Federal Institute for Risk AssessmentBerlin, Germany; ^2^German Center for Infection Research, Bundeswehr Institute of MicrobiologyMunich, Germany

**Keywords:** *Brucella*, phage, genome, virulent, lysotyping

## Abstract

Virulent phages have been used for many years to type *Brucella* isolates, but until recently knowledge about the genetic makeup of these phages remains limited. In this work the host specificity and genomic sequences of the original set (deposited in 1960) of VLA *Brucella* reference phages Tb, Fi, Wb, Bk2, R/C, and Iz were analyzed and compared with hitherto described brucellaphages. VLA phages turned out to be different from homonymous phages in other laboratories. The host range of the phages was defined by performing plaque assays with a wide selection of *Brucella* strains. Propagation of the phages on different strains did not alter host specificity. Sequencing of the phages Tb_V_, Fi_V_, Wb_V_, and R/C_V_ revealed nucleotide variations when compared to same-named phages previously described by other laboratories. The phages Bk2_V_ and Iz_V_ were sequenced for the first time. While Bk2_V_ exhibited the same deletions as Wb_V_, Iz_V_ possesses the largest genome of all *Brucella* reference phages. The duplication of a 301 bp sequence in this phage and the large deletion in Bk2_V_, Wb_V_, and R/C_V_ may be a result of recombination caused by repetitive sequences located in this DNA region. To identify new phages as potential candidates for lysotyping, the host range and Single Nucleotide Polymorphisms (SNPs) of 22 non-reference *Brucella* phages were determined. The phages showed lysis patterns different from those of the reference phages and thus represent novel valuable candidates in the typing set.

## Introduction

Brucellae are highly infectious and facultative intracellular bacterial pathogens causing brucellosis, a frequent zoonosis with more than 500,000 human cases reported worldwide every year (de Figueiredo et al., 2015). Infections may lead to reproductive failure and abortion in animals and a feverish multiorgan disease in humans. Up to now, 12 species were allocated to the genus *Brucella* (Godfroid et al., 2013). Six of them, *B. melitensis, B. abortus, B. suis, B. canis, B. ovis*, and *B. neotomae*, recovered from goats, cattle, pigs, dogs, sheep, and desert rats, respectively, belong to the “classical” or “historical” *Brucella* species (http://www.bacterio.net/brucella.html). Later on, “novel” *Brucella* species were isolated from cetaceans (*B. ceti*) and pinnipeds (*B. pinnipedialis*; Foster et al., 2007), voles (*B. microti*; Scholz et al., 2008b), baboons (*B. papionis*; Whatmore et al., 2014), red foxes (*B. vulpis*; Scholz et al., 2016) and from a human breast implant infection (*B. inopinata*; Scholz et al., 2010). Recently, a novel *Brucella* spp. reservoir in amphibians (e.g., Big-eyed tree frog: *Leptopelis verniculatus*; African bullfrogs: *Pyxicephalus edulis*; White's tree frog: *Litoria caerulea*) was discovered (Eisenberg et al., 2012; Fischer et al., 2012; Whatmore et al., 2015; Soler-Lloréns et al., 2016). Genetically, all *Brucella* species are closely related exhibiting genome similarities of >90% at the nucleotide level (Al Dahouk et al., 2010). Furthermore, because of the close genetic relationship of several genetic loci (e.g., 16S rRNA, 98.7% and *recA*, 85.5%) and a biochemical profile similar to *Ochrobactrum* spp., particularly atypical *Brucella* species like *B. microti* and *B. inopinata* are often misidentified using commercially available biochemical test systems (Scholz et al., 2008a,c).

Identification and subtyping of brucellae is time-consuming and laborious. Suspicious colonies on agar plates are primarily identified by a slide agglutination test using polyvalent *Brucella* antiserum (anti-S serum; Alton et al., 1975). Alternatively, PCR detection systems targeting the *bcsp31* gene and the intergenic sequence IS711 exist that are suited for the molecular detection of *Brucella* spp. (Baily et al., 1992; Hinic et al., 2008). Moreover, some multiplex PCR assays (e.g., AMOS, Bruceladder) were established for further species differentiation (López-Goñi et al., 2008; Mayer-Scholl et al., 2010). However, none of the available molecular typing systems cover all currently known species and biovars of the genus *Brucella*. In microbiological routine testing, the identification of species and biovars is based on specific properties of the bacteria (e.g., CO_2_ requirement, H_2_S production, urease activity, agglutination with monospecific A, R, and M sera, growth on media with thionin or basic fuchsin, metabolization of different substrates) and in particular susceptibility to lytic *Brucella* reference phages (Al Dahouk et al., 2010).

Phages which infect and lyse *Brucella* strains are known for over half a century (Parnas et al., 1958; Brinley-Morgan et al., 1960; Jablonski, 1962). After some basic characterization, a typing set comprising five reference phages [Tb (Tbilisi), Fi (Firenze), Wb (Weybridge), Bk (Berkeley), R/C] was developed (Corbel, 1984). Some years later the typing set was complemented by phage Iz (Izatnagar; Joint FAO/WHO Expert Committee on Brucellosis, 1986) and since then has been used in many diagnostic laboratories worldwide. The same holds true for a set of *Brucella* reference strains serving as controls for lysotyping. The original typing set has also been modified by adding other phages, e.g., S708, Bk2, F1, F25, and Np, some of which are mutants of the reference phages (Moreira-Jacob, 1968; Corbel et al., 1988; Rigby et al., 1989; Hammerl et al., 2014). All brucellaphages described so far have a podoviral morphology and are closely related, demonstrated by restriction analysis and southern hybridization (Segondy et al., 1988; Rigby et al., 1989). They are considered as a single taxonomic species (Corbel and Thomas, 1976; Ackermann et al., 1981). As a consequence, some phages possess an almost identical host specificity (Morgan, 1963; Calderone and Pickett, 1965). For a better identification and discrimination of isolates, *Brucella* lysotyping is mostly carried out by spot assays using both a routine test dilution (RTD), which is the highest dilution of a phage suspension producing confluent lysis of a propagator strain, and a 10,000 × RTD phage suspension. The main drawback of this procedure is that it cannot clearly distinguish between strains, in which the phages propagate, and those which are merely killed by the so-called *lysis from without* effect caused by a collapse of the cell wall in response to an overwhelming number of adsorbed phage particles (Corbel, 1984). Similar to growth inhibition, lysis from without is rather unspecific and more difficult to interpret than single plaques that unequivocally illustrate a phage infection (Jones et al., 1968). Furthermore, host range variants of *Brucella* reference phages have been isolated, particularly after changing the propagator strain (Corbel et al., 1988). Hence, it is not surprising that even studies, in which the same reference phages were tested, may show inconsistent results (Morris et al., 1973). For that reason it is important to know the biological and genetic properties of the typing phages exactly. First DNA sequences of phage Tb were reported by Zhu et al. (2009). The analysis of whole genome sequences of diagnostic brucellaphages confirmed their close relationship, even though phage Tb deposited in two different institutes revealed some sequence deviations, mainly point mutations (Flores et al., 2012; Farlow et al., 2014; Tevdoradze et al., 2015). Indeed, besides two InDels ~2.4 and 0.4 kb in size, the genomes of the investigated phages notably differ by single nucleotide polymorphisms (SNPs). Many of them were found in a gene probably encoding a tail collar protein, which has been suggested to be a determinant of host specificity. The importance of individual amino acids of the tail collar protein for the host range of the phages, however, has not yet been investigated.

In this work, the host specificity and genomes of six VLA *Brucella* reference phages (designated Tb_V_, Fi_V_, Wb_V_, Bk2_V_, R/C_V_, and Iz_V_ herein) were analyzed in detail. The genome sequences of Bk2_V_ and Iz_V_ will be presented for the first time. The phages were compared with homonymous phages originating from other laboratories. Bioinformatic analyses revealed DNA repeats within the phage genomes, which may be important for the acquisition, loss or duplication of DNA sequences. The host range determination of 22 non-reference phages disclosed some potential candidates useful for lysotyping.

## Materials and methods

### Bacterial strains, media, and growth conditions

Detailed information on all strains used in this study is given in Table S1. Cultivation of the bacteria was performed as previously described (Alton et al., 1975). Solid and overlay agar contained 1.8 and 0.7% (w/v) bacto-agar No. 1 (Oxoid, Wesel, Germany), respectively.

### Propagation of bacteriophages

Relevant data on brucellaphages used in this study are given in Table S2. The reference phages Tb_V_, Bk2_V_, Wb_V_, Fi_V_, R/C_V_, and Iz_V_ were obtained from the Weybridge bacteriophage collection in form of unopened lyophilized phage stocks produced by the OIE Brucellosis Reference Centre of the Veterinary Laboratories Agency (VLA, Addlestone, UK) in 1973. Lyophilized phages were suspended in 5 ml of SM-buffer (100 mM NaCl, 8 mM MgSO_4_ 7H_2_O, 50 mM Tris-HCl, pH 7.5). The suspensions were used for plaque assays by the softagar overlay method as previously described (Sambrook and Russell, 2001). In contrast to phage R/C_V_, which was propagted on *B. ovis* strain 63/290, Tb_V_, Fi_V_, Bk2_V_, Wb_V_, and Iz_V_ were propagated on *B. abortus* vaccine strain S19. Besides the standard reference phages which are globally used for *Brucella*-typing, other brucellaphages were included in the study. While lyophilized A422 and M51 stocks from 1960 were also provided by VLA, the origin of the phages F1, F1m, F1u, F25, F25u, F44, F45, F48, FO1, P, P2, 3, 6, 7, 10I, 12m, 24II, 45II, 212XV, and 371XXIX is unknown. Lyophilized stocks of these phages prepared at the BfR in 1973 were used for further investigation. However, there is no information available on how these phages were propagated before lyophilization. To ensure the purity of all brucellaphages, a three-fold successive single plaque separation was performed. After the third plaque separation purified phages were used for the preparation of high titer lysates (>10^9^ PFU/ml). To accomplish this, 10^6^ PFU were applied to 20 ml of an early logarithmic growing (McFarland 1.0–1.5) *Brucella* culture followed by incubation for 24–48 h on a rotational shaker (100 rpm) under microaerobic conditions. Thereafter, lysates were centrifuged for 10 min at 7,500 × g to remove cellular debris. Supernatants were subjected to sterile filtration (0.45 and 0.2 μm; Merck Millipore, Schwalbach, Germany) and DNaseI-/RNaseA-treatment (10 μg/ml wt/vol each; Roche, Mannheim, Germany). Phage particles were concentrated and purified by discontinuous CsCl-step gradients (CsCl, 1.3 to 1.7 g ml^−1^) as described previously (Sambrook and Russell, 2001). Phage bands recovered from CsCl-gradients were desalted using 100K Amicon Ultra centrifugal filter columns (Merck Millipore).

### Host range determination

Host range analyses were carried out by spot assays on *Brucella* spp. reference and type strains (*n* = 26; Table S1) and field isolates as well as reference strains of *Ochrobactrum* spp. (*n* = 119), *Yersinia enterocolitica* O:9 (*n* = 7), *Mesorhizobium* sp. (*n* = 6), *Sinorhizobium meliloti* (*n* = 5), and *Pseudomonas* (*n* = 5; data not shown). Two hundred microliters of each strain were mixed with 5 ml of pre-warmed *Brucella-*broth soft agar (0.7%) and poured onto a lysogeny-broth (LB) agar plate. Ten microliter aliquots of 1:10 serial dilutions of each lysate were spotted onto the overlay agar. Agar plates were visually inspected after incubation for 24 and 48 h at 37°C. Phages that did not affect bacterial growth were classified as non-infectious (negative: −). Phages were classified as infectious (positive: +) when single plaques were identified in spotting zones of the tested lysates. In case of growth inhibition (GI) visible by an even but decreased bacterial growth within the spotting areas, the respective phage/strain combinations were further investigated by preparing phage lysates. For this purpose, 10^7^ phages were applied to 20 ml of an early exponential growing *Brucella* culture (McFarland: 1.0–1.5). After incubation at 37°C for 48 h, lysates were purified as described above. Propagation of phages was determined by plaque assays (Sambrook and Russell, 2001). Four hundred microliters of a *Brucella* culture (McFarland: 5.0–7.0) were mixed with 100 μl aliquots of 1:10 serial dilutions of each lysate, incubated for 20 min at room temperature, added to 5 ml of pre-warmed *Brucella-*broth soft agar (0.7%) and poured onto LB agar plates. After incubation for 24 and 48 h at 37°C, agar plates were visually inspected for plaque activity. If phage replication occurred, the lysates contained more than 10^7^ infectious particles. Strains which increased the phage titer were finally classified as susceptible (positive: +).

### Isolation of phage DNA, whole genome sequencing, and bioinformatic analysis

Phage DNA extraction from CsCl-purified particles was performed as previously described (Hammerl et al., 2016). Determination of phage genomic sequences was conducted using a Roche 454 genome sequencer FLX titanium system by GATC Biotech AG (Konstanz, Germany). Library generation and 454 FLX sequencing were carried out according to the procedure of the manufacturer (Roche/454 Life Sciences, Branford, Connecticut, USA). Sequence reads were assembled using the Roche/454 Newbler software at default settings (454 Life Sciences Corporation, Software release 2.3) resulting in one contig with an average sequence coverage of >100 per consensus base. Sequence analysis and alignments were carried out using Accelrys Gene v2.5 (Accelrys Inc., San Diego, CA, USA). Bioinformatic analysis and genome annotation were performed as described previously (Hammerl et al., 2014, 2016).

### PCR analysis

PCR was performed in an Eppendorf Mastercycler ep Gradient (Eppendorf, Hamburg, Germany) according to standard protocols. Single reactions were carried out with ~10 ng/μl phage DNA and 2.5 μl of each primer in a final volume of 50 μl using Qiagen DNA polymerase amplification components (Qiagen, Hilden, Germany). For PCR amplification the following parameters were used: initial template denaturation at 96°C for 120 s followed by 35 cycles including denaturation at 96°C for 15 s, annealing at 55°C for 5 min and elongation for 210 s at 72°C. A final elongation step at 72°C for 1 min was added. Purification of PCR products was performed by using the MSB spin PCRapace kit (Stratec, Birkenfeld, Germany). The nucleotide sequence of the PCR products was determined by Sanger sequencing (Eurofins Genomics, Ebersberg, Germany).

### Nucleotide sequence accession number

The complete nucleotide sequences of the brucellaphages were submitted to GenBank under the accession numbers HF569092 (Wb_V_), HF569091 (Tb_V_), HF569089 (Fi_V_), HF569091 (Bk2_V_), HF569090 (R/C_V_), and KY056619 (Iz_V_).

## Results and discussion

### Host range determination of the VLA *Brucella* reference phages

A comprehensive study with 10 *Brucella* species represented by 26 reference and type strains was performed to elucidate in which strains the group I to group VI *Brucella* reference phages Tb_V_, Fi_V_, Wb_V_, Bk2_V_, R/C_V_, and Iz_V_ (Corbel, 1987) replicate and which strains are merely lysed by lysis from without at high MOIs. Lytic activity of the phages was quantified by plaque assays (titration) allowing an accurate determination of the hosts' susceptibility. If no plaque formation occurred and only growth inhibition was observed, we examined propagation of the phages using liquid cultures of the respective strains (see section Materials and Methods). The phages Tb_V_ (group I) and Fi_V_ (group II) showed almost identical lysis patterns (Table 1). They infected reference strains of the eight *B. abortus* biovars (bv1–7, 9), *B. neotomae* 5K33, *B. microti* CCM4915 and two strains of the recently identified species *B. vulpis* (Scholz et al., 2016). On the other hand, *B. suis* 1,330 (bv1) was only lysed by phage Tb_V_, but not by Fi_V_. Data that have been published on the susceptibility of *B. suis* 1,330 to phage Tbilisi are rather contradictory. While several articles reported that this strain was not lysed by the phage at RTD (Jones et al., 1968; Flores et al., 2012), other authors obtained plaques with both Tbilisi and Firenze (Rigby et al., 1989). Another deviation from published data concerns *B. melitensis* 16M (bv1), for which we and also others could not confirm infection by Tbilisi and Firenze, whereas Rigby et al. (1989) reported on plaque formation caused by these phages (Rigby et al., 1989). This raises the question about the reasons for these discrepancies? One possible explanation is that in our study and in the study of Rigby et al. (1989), phages were propagated on the *B. abortus* vaccine strain S19 (bv1), which makes host adaptation as a reason for different lysis patterns improbable. Furthermore, the slightly different methodologies applied to determine lysis patterns might not account for diverging results. It is more likely that the phages and/or indicator strains used in the two studies were actually not identical, perhaps due to mutations in genes important for phage infection. Of course, this presumption can only be confirmed by whole genome sequencing of the used strains and phages. For phage Tbilisi different variants (Tb_M_ and Tb_Y_) have already been described (Foster et al., 2007; Flores et al., 2012). In addition, it has been demonstrated that the propagation of brucellaphages on different indicator strains caused changes in the phage genome, which might alter host specificity (Tevdoradze et al., 2015). However, lysis patterns of the phages have not yet been determined and compared. We therefore address this issue in detail.

**Table 1:** **Host range determination of ***Brucella*** reference phages**.

***Brucella* spp. (Strain)**	***Brucella* phage group**					
	**I**	**II**	**III**	**IV**	**V**	**VI**
	**Wb_V_**	**Fi_V_**	**Bk2_V_**	**Tb_V_**	**R/C_V_**	**Iz_V_**
***B. abortus***	+	+	+	+	−	+
*B. abortus* (S19)	2 × 10^9^	2 × 10^9^	2 × 10^9^	2 × 10^9^	NL	2 × 10^9^
*B. abortus* bv1 (544)	7 × 10^7^	4 × 10^7^	2 × 10^8^	2 × 10^8^	NL	6 × 10^7^
*B. abortus* bv2 (86/8/59)	1 × 10^8^	1 × 10^8^	1 × 10^9^	1 × 10^8^	NL	3 × 10^8^
*B. abortus* bv3 (Tulya)	1 × 10^8^	6 × 10^6^	1 × 10^7^	2 × 10^8^	NL	3 × 10^7^
*B. abortus* bv4 (292)	2 × 10^8^	6 × 10^6^	1 × 10^7^	3 × 10^7^	NL	3 × 10^7^
*B. abortus* bv5 (B3196)	1 × 10^8^	1 × 10^7^	1 × 10^8^	1 × 10^7^	NL	1 × 10^7^
*B. abortus* bv6 (870)	6 × 10^7^	6 × 10^7^	1 × 10^7^	2 × 10^7^	NL	3 × 10^7^
*B. abortus* bv7 (63/75)	4 × 10^7^	1 × 10^6^	3 × 10^8^	7 × 10^7^	NL	7 × 10^6^
*B. abortus* bv9 (C68)	8 × 10^7^	2 × 10^7^	3 × 10^7^	3 × 10^7^	NL	2 × 10^7^
***B. melitensis***	±	−	+	−	−	+
*B. melitensis* bv1 (16M)	1 × 10^7^	NL	1 × 10^8^	NL	NL	1 × 10^8^
*B. melitensis* bv2 (63/9)	NL	NL	1 × 10^7^	NL	NL	5 × 10^6^
*B. melitensis* bv3 (Ether)	3 × 10^7^	NL	6 × 10^7^	NL	NL	5 × 10^7^
***B. suis***	±	−	±	±	−	±
*B. suis* bv1 (1330)	1 × 10^9^	NL	2 × 10^8^	5 × 10^7^	NL	2 × 10^8^
*B. suis* bv2 (Thomsen)	GI	NL	GI	GI	NL	GI
*B. suis* bv3 (686)	1 × 10^7^	NL	3 × 10^7^	NL	NL	3 × 10^7^
*B. suis* bv4 (40)	5 × 10^8^	NL	4 × 10^8^	NL	NL	3 × 10^8^
*B. suis* bv5 (513)	5 × 10^8^	NL	5 × 10^8^	NL	NL	2 × 10^8^
***B. ovis***	−	−	−	−	+	−
*B. ovis* (63/290)	NL	NL	NL	NL	2 × 10^8^	NL
***B. neotomae***	+	+	+	+	−	+
*B. neotomae* (5K33)	1 × 10^9^	2 × 10^7^	2 × 10^8^	1 × 10^8^	NL	1 × 10^8^
***B. canis***	−	−	−	−	−	−
*B. canis* (RM6/66)	NL	NL	NL	NL	1 × 10^7^	NL
***B. ceti***	−	−	−	−	−	−
*B. ceti* (B1/94)	GI	GI	GI	GI	NL	GI
***B. pinnipedialis***	+	−	+	−	−	−
*B. pinnipedialis* (B2/94)	4 × 10^6^	NL	2 × 10^6^	NL	NL	NL
***B. microti***	+	+	+	+	−	+
*B. microti* (CCM 4915)	1 × 10^9^	2 × 10^5^	5 × 10^8^	2 × 10^7^	NL	2 × 10^8^
***B. inopinata***	−	−	−	−	−	−
*B. innopinata* (BO1)	NL	NL	NL	NL	NL	NL
***B. vulpis***	+	+	+	+	−	+
*B. vulpis* (FH60HL)	2 × 10^9^	3 × 10^7^	1 × 10^9^	2 × 10^8^	NL	5 × 10^8^
*B. vulpis* (FH965HL)	1 × 10^9^	2 × 10^8^	1 × 10^9^	4 × 10^8^	NL	2 × 10^8^

*+, Plaque formation, −, no plaque formation. bv, biovar; NL, no lysis; GI, growth inhibition*.

Unlike Tb_V_ and Fi_V_, the phages Wb_V_ (group III), Bk2_V_ (group IV), R/C_V_ (group V), and Iz_V_ (group VI) revealed lysis patterns that correlated well with published data. The species *B. ceti* and *B. inopinata* were not infected by any phage. Five phages inhibited the growth of the *B. ceti* reference strain but replication of the phages did not occur. Though, a number of *B. ceti* strains isolated from cetaceans were lysed by Weybridge and Izatnagar in another study (Foster et al., 2007). In contrast, for *B. inopinata* which is currently represented only by its type strain BO1, no lytic phage has been found so far. Since this strain harbors an active prophage (Hammerl et al., 2016) that might affect the susceptibility to other phages, the importance of the prophage was analyzed in more detail (see next chapter). To avoid misinterpretation of phage typing results, plaque assays allowing a quantitative determination of the lytic activity are certainly better suited than spot assays using high titer lysates, since lysis from without effects are too variable to identify species unequivocally. We also studied infection of other species (*Ochrobactrum* spp., *Mesorhizobium* sp., *Sinorhizobium meliloti, Yersinia enterocolitica* O:9, *Pseudomonas* sp.) by the brucellaphages and detected growth inhibition of some strains (data not shown). As the zones of growth inhibition looked similar to halos obtained with *Brucella*, such results could be misleading.

### Propagation of the phages on alternative strains did not alter lysis patterns

In this set of experiments the question should be answered whether lytic specificity of the phages may be affected by a change of propagator strains. For that reason Tb_V_, Wb_V_, R/C_V_, and Iz_V_ were co-cultivated with *B. abortus* S19, *B. melitensis* 16M, *B. suis* 1,330 and *B. ovis* 63/290. After overnight incubation, phages were isolated and used for the next co-cultivation with the respective strain. This procedure was repeated 20 times corresponding to ~500 generations. Thereafter, host ranges of the phages were examined by plaque assays testing all reference and type strains (Table S1). Following this procedure, no change of lysis patterns was detected. Furthermore, no adaptation of the phages to new hosts was observed. Phage R/C_V_ e.g., remained its specificity and infected exclusively rough strains. The data suggest that even though propagation of brucellaphages on different strains may cause genomic changes (Tevdoradze et al., 2015) this is not necessarily associated with an alteration of the host range. However, the number of phage particles released from individual strains can differ significantly (data not shown). Thus, lysates of the same phage exhibited different titers which may bias a result. To avoid diverging lysis patterns, we recommend to propagate diagnostic brucellaphages on the same indicator strain and to examine the phage genomes by sequencing if results are inconsistent.

Since many phages have been isolated from *Brucella* cultures, a lysogenic state termed pseudolysogeny has been suggested for these phages (Renoux and Suire, 1963). Lysogeny may influence the susceptibility of the bacteria to phages. We investigated *B. abortus* S19 colonies that had survived infection by phage Tb_V_. The isolated colonies were passaged several times. While a release of phage particles after mitomycin C treatment (Hammerl et al., 2016) was not observed, Tb_V_ was identified in initial cultures by PCR. In addition, electron microscopy revealed Tb_V_ particles adsorbed to the cell wall (data not shown). Even after repeated cultivation, the phage was no longer detectable. The data indicate that strain *B. abortus* S19 may serve as carrier for Tb_V_ but that there is obviously no integration of the phage genome into the bacterial chromosomes. We also did not observe any immunity of the Tb_V_-carrying bacteria against superinfection by the same or other *Brucella* reference phages. Similar results were published by other authors (Morris et al., 1973; Corbel and Morris, 1975). To elucidate whether Tb_V_-induced cell lysis may be affected by endogenous prophages providing immunity to superinfection, a S19 derivative (S19lys) containing the temperate phage BiPBO1 (Hammerl et al., 2016) was studied. Tb_V_ lysed the lysogenic strain like the original strain without the prophage. Thus, BiPBO1 did not affect Tb_V_ propagation. However, lysates prepared with strain S19lys contained both Tb_V_ and BiPBO1 which could be easily identified by their different plaque morphologies. As the BiPBO1 prophage was induced by infection with Tb_V_, lysogeny has to be taken into account when lysates of brucellaphages are prepared. Otherwise, incorrect results might be obtained when the phages are used for typing.

### HindIII restriction analysis is suited to allocate Bk2_V_ and Iz_V_ to existing phage groups

**Figure 1:**
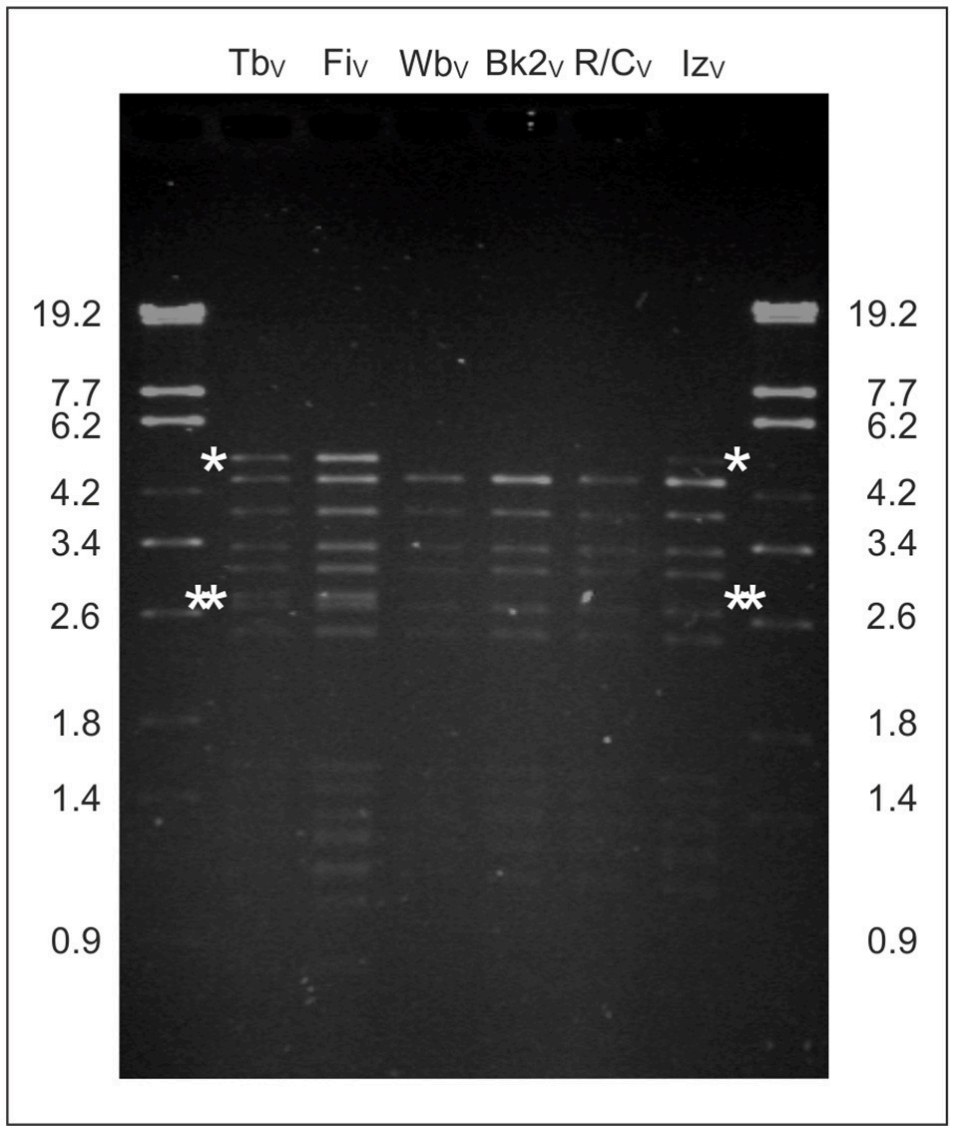
**HindIII restriction analysis of the VLA ***Brucella*** reference phages**. Fragments that are present in Tb_V_, Fi_V_, and Iz_V_ but absent in Wb_V_, Bk2_V_, and R/C_V_ are marked by asterisks.

Previous studies on the *Brucella* reference phages Tbilisi, Weybridge, Bk2, and R/O (an instable variant of phage R/C) showed that their genomes cannot be distinguished by restriction analysis using the endonucleases BamHI, EcoRI, and PvuII, because identical fragment patterns were obtained (Segondy et al., 1988). In contrast, phage Nepean (Np) revealed some differences, e.g., an additional 1.0 kb fragment in the HindIII digest (Rigby et al., 1989). The hitherto sequenced brucellaphage genomes mainly differed by two major InDels and can thus be assigned to two groups (Flores et al., 2012; Farlow et al., 2014; Tevdoradze et al., 2015). The presence or absence of these sequences should be traceable by use of suitable restriction enzymes. We analyzed HindIII restriction patterns of the VLA reference phages in detail to ascertain whether the yet not sequenced phages Bk2_V_ and Iz_V_ belong to one of the existing groups. As documented in Figure 1, phage Tb_V_ and Fi_V_ showed two additional restriction fragments, 5.0 kb and 2.8 kb in size, which were absent in Wb_V_ and R/C_V_. These fragments comprise DNA sequences that are missing in the latter phages. The fragments were also absent in the Bk2_V_ restriction digest. This suggests that Bk2_V_ may exhibit identical deletions as Weybridge and R/C. The two above mentioned restriction fragments of Tb_V_ and Fi_V_ were detected in Iz_V_ indicating that this phage resembles Tb_V_ and Fi_V_ in this respect. In summary, restriction analysis using HindIII is a fast and easy method to determine whether a new phage contains deletions similar to already known brucellaphages.

### The VLA *Brucella* reference phages are not identical to same-named phages in other laboratories

Host range analyses showed that the six VLA reference phages infected a distinct range of strains, even though some phages revealed almost identical lysis patterns. The only difference that was observed between Tb_V_ and Fi_V_ was *B. suis* strain 1,330 that was infected by Tb_V_ but not by Fi_V_. Similarly, the host range of Wb_V_, Bk2_V_ and Iz_V_ only differed by one or two strains (Table 1). To allow a comparison between host specificity and genome variations, the phages were sequenced, two of them (Bk2_V_ and Iz_V_) for the first time. The remaining four phages (Tbilisi, Firenze, Weybridge, and R/C) have already been characterized in previous studies but none of the genomic sequences determined in this work was identical to those described by other authors. Tb_V_ showed eight SNPs compared to phage Tb_W_ deposited at the Félix d'Hérelle Reference Center for Bacterial Viruses, Université Laval, Canada (Farlow et al., 2014). Five SNPs are silent mutations, two others lying within ORF21 (neck protein) and ORF44 (hypothetical protein) caused an amino acid exchange and one (within ORF14) is an insertion resulting in a truncated protein of unknown function. All these SNPs plus 14 additional SNPs exist when Tb_V_ is compared with Tb_E_ isolated at Eliava, Tbilisi, Ukraine (Tevdoradze et al., 2015). Differences to Tb_M_ (Gamaleya Scientific Research Institute of Epidemiology and Microbiology, Moscow, Russia) are even more pronounced and have already been addressed by Farlow et al. (2014). Contrary to Tb_V_, Fi_V_ exhibited only two SNPs compared to Firenze in Laval. One SNP is located in an intergenic region, the other SNP caused an amino acid exchange at the C-terminus of a hypothetical protein. Wb_V_ showed five single nucleotide deviations to its counterpart in Laval. Three of them are located in genes (ORF16 and ORF23) for structural proteins, one in ORF27 probably encoding a tail collar protein and one in ORF57 for a primase/DNA polymerase. All of them caused amino acid exchanges. The most pronounced discrepancies between two phages with identical designations were found in R/C. The genomic sequence of the VLA R/C phage strain is 45 bp shorter than that of R/C in Laval. Furhermore, nine SNPs and two InDels were identified. Most deviations (four amino acid exchanges and one deletion of two amino acids) were found in the tail collar protein. Two frame shift mutations leading to radically changed gene products are present in ORF11 and ORF14 encoding a hypothetical protein and a primase/DNA polymerase, respectively. Quite the opposite was observed for Bk2_V_. The host range of this phage differs significantly from that of Bk (Corbel, 1987; Farlow et al., 2014) but on the genome only one SNP located in the tail collar protein gene leading to an amino acid exchange was detected.

**Figure 2:**
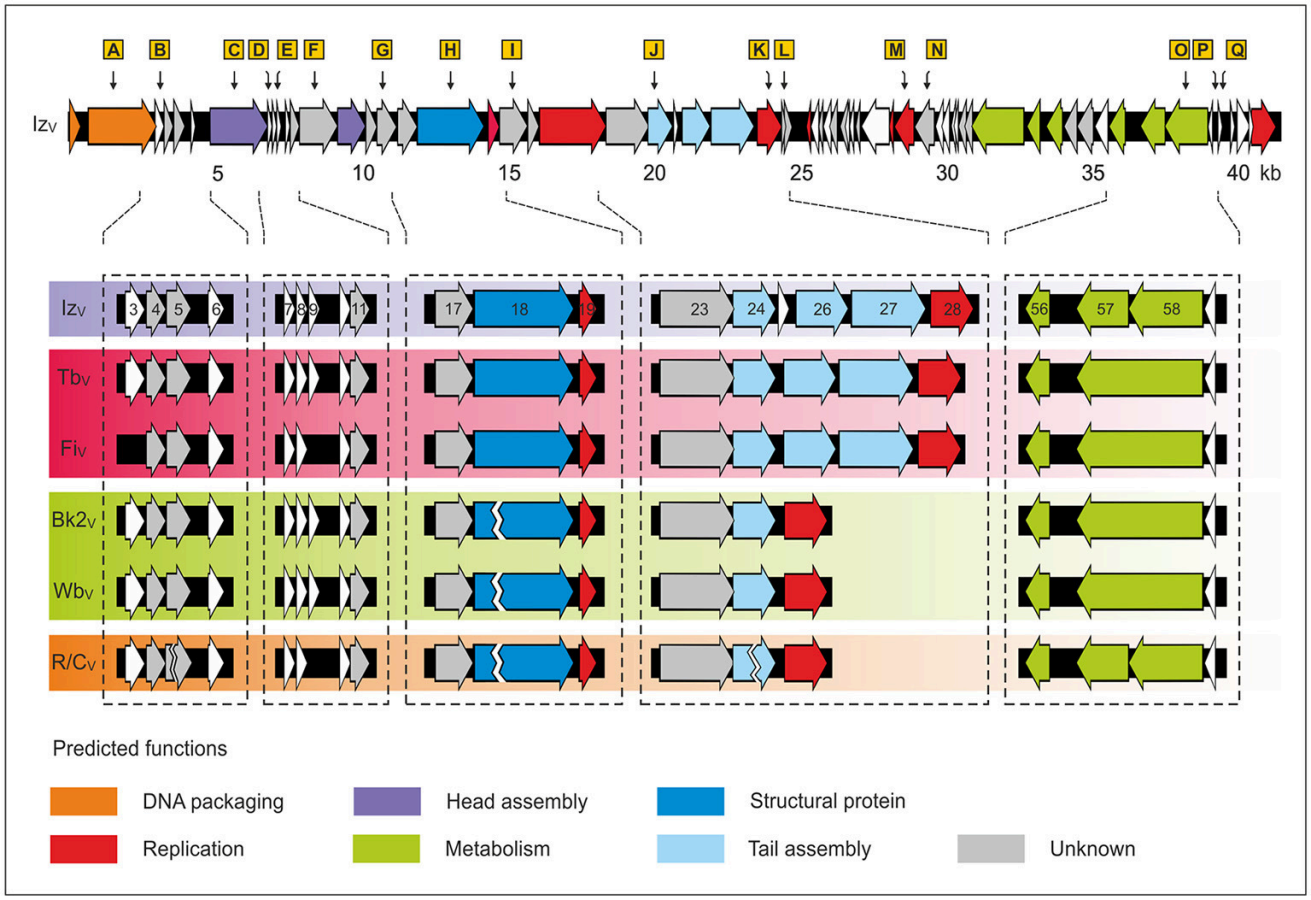
**Genome organization of the VLA ***Brucella*** reference phages**. The upper panel shows the gene map of Iz_V_. Putative genes are colored according to the predicted functions of their gene products Table S4. SNPs identified by comparison with the other reference phages are indicated by orange rectangles (A–Q). For a better overview, only one SNP per gene is shown. A list with all SNPs is presented in Table S3. The lower panel illustrates the gene composition of the six VLA reference phages. Relevant genes are numbered. In Wb_V_, Bk2_V_ and R/C_V_ ORF27 and ORF28 are deleted, while ORF18 exhibits only are partial deletion. In Iz_V_ and R/C_V_ ORF57 is splitted into two ORFs Iz_V_ additionally contains the novel ORF 25 generated by sequence duplication.

Phage Iz_V_ exhibits the largest genome (41,446 bp) of all hitherto described brucellaphages. It contains a 301 bp duplicated nucleotide sequence located between the ORFs 23 and 24, which code for tail fiber proteins. Apart from this deviation, the Iz_V_ genome composition is similar to those of Tb_V_ and Fi_V_ as it does not carry the two deletions present in other *Brucella* reference phages (Figure 2). However, based on the SNP data the closest relative of Iz_V_ is not Tb_V_ or Fi_V_, but phage Bk2_V_. Besides the two InDels there are only five SNPs in these two phages. All of the SNPs are similarly present in the phages Tb_V_, Fi_V_, Wb_V_, and R/C_V_. The SNPs are located in genes for the large terminase subunit, neck protein, a hypothetical protein and the primase/DNA polymerase (Table S3). Tb_V_ and Fi_V_ revealed 17 additional SNPs which are spread all over the phage genomes (Figure 2). Another interesting feature of Iz_V_ is that the gene for the primase/DNA polymerase contains an internal stop codon, caused by the deletion of a single nucleotide. Therefore, in Iz_V_ the largest gene of brucellaphages is splitted into two smaller ORFs. While the primase/DNA polymerase of Tb_V_, for instance, comprises 780 amino acids, the ORFs 57 and 58 of Iz_V_ encode polypeptides of 496 and 284 amino acids. Because of the frame shift mutation, the eight C-terminal amino acids of the large Iz_V_ polypeptide diverge from the Tb_V_ protein. The small polypeptide exhibits no differences to the Tb_V_ sequence. Our analysis of the R/C_V_ genomic sequence revealed that this phage also contains a stop codon within the primase/DNA polymerase gene. In this phage polypeptides of 236 and 555 amino acids are encoded. The data demonstrate that several variants of the primase/DNA polymerase exist in brucellaphages. Whether the two proteins of Iz_V_ and R/C_V_ possess the same activity as their larger counterpart in Tb_V_ is unknown and has to be clarified by further experiments.

On the basis of host range and whole genome analyses, Farlow et al. (2014) divided the Laval reference phages into three groups. Group I is composed of Tbilisi and Firenze, group II includes Berkeley, R/C and Pr from Mexico and group III contains Weybridge and S708. Some of these phages were also investigated in this study. However, since the VLA and Laval reference phages are not identical, they cannot be easily compared. From the data obtained in our study, the VLA reference phages can be allocated to four groups. As in the classification mentioned above, Tb_V_ and Fi_V_ belong to group I. Group II consists of Wb_V_ and Bk2_V_, which exhibited an almost identical host range and which are genetically closely related. Phage R/C_V_ represents group III. It possesses an unique host specificity and showed some deletions and frame shift mutations not occurring in the other reference phages. The fourth group is represented by Iz_V_ which does not fit to the other groups, neither by its host range, nor in terms of its genomic sequence. Due to the close overall DNA homologies of brucellaphages and because nucleotide variations can be observed after changing the host strain (Tevdoradze et al., 2015), the question arises, whether it really makes sense to group these phages. In addition, it should be considered that *Brucella* strains having the same designation do not need to be identical. This also can distort results, e.g., the determination of the host range of the phages.

### Identification of repetitive DNA sequences possibly involved in genomic rearrangements

**Figure 3:**
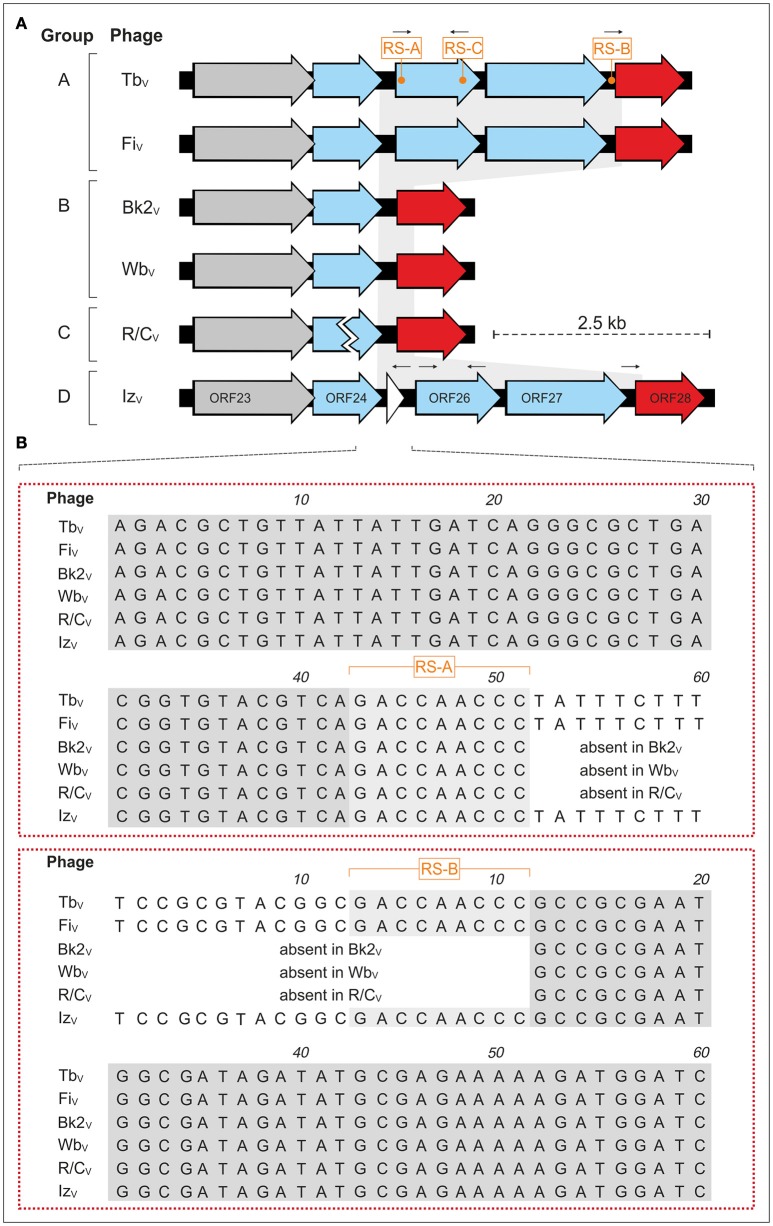
**Analysis of the large (2.443 bp) InDel in ***Brucella*** reference phages. (A)** Gene arrangement in this DNA region. The position of the repeats RS-A, RS-B, and RS-C and of the additional inverted repeat present in Iz_V_ are indicated. **(B)** Alignment of the sequences flanking the InDel. The DNA fragment deleted in Wb_V_, Bk2_V_, and R/C_V_ borders on the 9 bp direct repeats RS-A and RS-B.

Sequence determination of *Brucella* reference phages disclosed a 2.4 kb DNA fragment comprising two genes for tail fiber proteins present in Tb_V_, Fi_V_, and Iz_V_, but absent in Wb_V_, Bk2_V_, and R/C_V_ (Figure 3). Subsequent InDel analysis revealed some remarkable consistencies among various phages. Compared to the other reference phages, Wb_V_, Bk2_V_, and R/C_V_ show exactly the same deletion of 2,443 bp. In Tb_V_, Fi_V_, and Iz_V_, the fragment is flanked by a 9 bp direct repeat termed RS-A and RS-B (5′-GACCAACCC-3′, Figure 3). A third copy of this sequence (RS-C) exists in reverse complement orientation ~700 bp apart from RS-A. By contrast, the Wb_V_, Bk2_V_, and R/C_V_ genomes contain only one copy of this sequence, adjacent to the deleted fragment. Notably, the 301 bp sequence that is duplicated in Iz_V_ also borders with one end on RS-A. At the other end of the duplicated sequence, a similar motif (5′-ACCAAACCC-3′) is located in reverse complement orientation (Figure 3). This sequence does not exist in the other VLA *Brucella* reference phages. The duplication resulted in the generation of the new ORF 25 in Iz_V._ These data suggest that the identified repeats may be important for the acquisition, loss or duplication of DNA sequences in brucellaphages. The additional 1.0 kb HindIII fragment identified in phage Nepean but not in other reference phages has also been suggested to be a repetition as it hybridized to Tbilisi DNA (Tevdoradze et al., 2015). It would be interesting to learn whether the repetition in this phage is similarly flanked by the repeats described above.

### New phages may be helpful to improve the typing set

This study, and also those of other authors revealed several difficulties that may arise, when certain *Brucella* strains are typed using the existing reference phage typing set. The main reasons for this are the very similar host ranges of the phages and the fact that lysis from without effects, which are more difficult to interpret than plaques, have yet been included in the evaluation of lysis patterns. In addition, there are apparently host range variants of the reference phages, which further exacerbate the situation because it makes the comparison of data collected in different laboratories difficult. We therefore determined the host range of 22 non-reference phages deposited in the *Brucella* phage collection of the BfR (Table S2) to identify further candidates for typing. The analysis disclosed a very similar host specificity of the phages (Table 2). Though, the host range was clearly different from those of the reference phages. Like Tb_V_ and Fi_V_, none of the phages lysed strains of *B. melitensis* and *B. pinnipedialis*. All phages infected at least two, most of them even three *B. suis* biovars, namely bv1, bv4, and bv5. In this regard, the phages resemble Wb_V_, Bk2_V_, and Iz_V_. Lysis of *Brucella* species was similar to the reference phages. The exact analysis of the phages' host range also revealed some individual differences. This particularly pertained to the *B. suis* bv5 strain 513 that was not lysed by five phages. *Brucella microti* CCM4915^T^ was resistant only to one phage. Besides these specificities, all phages infected the same strains. It should, however, be emphasized that the phages produced a reduced number (up to 4 log units) of plaques on *B. suis* and *B. microti* strains. This has to be considered when the phages are used for typing. Two of the non-reference phages (A422 and M51) have been already studied by Morris et al. (1973). While we found plaques on *B. suis* with both phages, the other authors reported on lytic activity at RTD only for M51. It is conceivable that their A422 lysate did not contain enough active phage particles to cause lysis at RTD. Nevertheless, in spite of this discrepancy, the data of this study suggest that the reference phage typing set could be complemented by additional phages, which would make the discrimination of some *Brucella* species and strains more reliable.

**Table 2:** **Host range of other brucellaphages**.

***Brucella strain***	**F44**	**P**	**45 III**	**FO1**	**F1m**	**F1u**	**F25u**	**F45**	**F48**	**3**	**6**	**7**	**10I**	**12m**	**24II**	**212 XV**	**371 XXIX**	**P2**	**M51**	**A 422**	**F25**	**F1**
*B. abortus* 544	+	+	+	+	+	+	+	+	+	+	+	+	+	+	+	+	+	+	+	+	+	+
*B. abortus* 86/8/59	+	+	+	+	+	+	+	+	+	+	+	+	+	+	+	+	+	+	+	+	+	+
*B. abortus* Tulya	+	+	+	+	+	+	+	+	+	+	+	+	+	+	+	+	+	+	+	+	+	+
*B. abortus* 292	+	+	+	+	+	+	+	+	+	+	+	+	+	+	+	+	+	+	+	+	+	+
*B. abortus* B3196	+	+	+	+	+	+	+	+	+	+	+	+	+	+	+	+	+	+	+	+	+	+
*B. abortus* 870	+	+	+	+	+	+	+	+	+	+	+	+	+	+	+	+	+	+	+	+	+	+
*B. abortus* 63/75	+	+	+	+	+	+	+	+	+	+	+	+	+	+	+	+	+	+	+	+	+	+
*B. abortus* C86	+	+	+	+	+	+	+	+	+	+	+	+	+	+	+	+	+	+	+	+	+	+
*B. melitensis* 16M	−	−	−	−	−	−	−	−	−	−	−	−	−	−	−	−	−	−	−	−	−	−
*B. melitensis* 63/9	−	−	−	−	−	−	−	−	−	−	−	−	−	−	−	−	−	−	−	−	−	−
*B. melitensis* Ether	−	−	−	−	−	−	−	−	−	−	−	−	−	−	−	−	−	−	−	−	−	−
*B. suis* 1330	(+)	(+)	(+)	(+)	(+)	(+)	(+)	(+)	(+)	(+)	(+)	(+)	(+)	(+)	(+)	(+)	(+)	(+)	(+)	(+)	(+)	(+)
*B. suis* Thomsen	−	−	−	−	−	−	−	−	−	−	−	−	−	−	−	−	−	−	−	−	−	−
*B. suis* 686	−	−	−	−	−	−	−	−	−	−	−	−	−	−	−	−	−	−	−	−	−	−
*B. suis* 40	(+)	(+)	(+)	(+)	(+)	(+)	(+)	(+)	(+)	(+)	(+)	(+)	(+)	(+)	(+)	(+)	(+)	(+)	(+)	(+)	(+)	−
*B. suis* 513	−	(+)	−	(+)	(+)	(+)	(+)	(+)	(+)	(+)	−	(+)	(+)	(+)	(+)	(+)	(+)	(+)	(+)	−	−	(+)
*B. canis* RM 6/66	−	−	−	−	−	−	−	−	−	−	−	−	−	−	−	−	−	−	−	−	−	−
*B. neotomae* 5K33	+	+	+	+	+	+	+	+	+	+	+	+	+	+	+	+	+	+	+	+	+	+
*B. ovis* 63/290	−	−	−	−	−	−	−	−	−	−	−	−	−	−	−	−	−	−	−	−	−	
*B. ceti* B1/94	−	−	−	−	−	−	−	−	−	−	−	−	−	−	−	−	−	−	−	−	−	
*B. pinnipedialis* B2/94	−	−	−	−	−	−	−	−	−	−	−	−	−	−	−	−	−	−	−	−	−	
*B. microti* CCM4915^T^	(+)	(+)	(+)	(+)	(+)	(+)	(+)	(+)	(+)	(+)	−	(+)	(+)	(+)	(+)	(+)	(+)	(+)	(+)	(+)	(+)	(+)
*B. inopinata* BO1	−	−	−	−	−	−	−	−	−	−	−	−	−	−	−	−	−	−	−	−	−	−
*B. vulpis* F60 H	+	+	+	+	+	+	+	+	+	+	+	+	+	+	+	+	+	+	+	+	+	+
*B. vulpis* F965 H	+	+	+	+	+	+	+	+	+	+	+	+	+	+	+	+	+	+	+	+	+	+

*+, Plaque formation; (+), reduced plaque formation in comparision to B. abortus strain S19; −, no plaque formation*.

**Figure 4:**
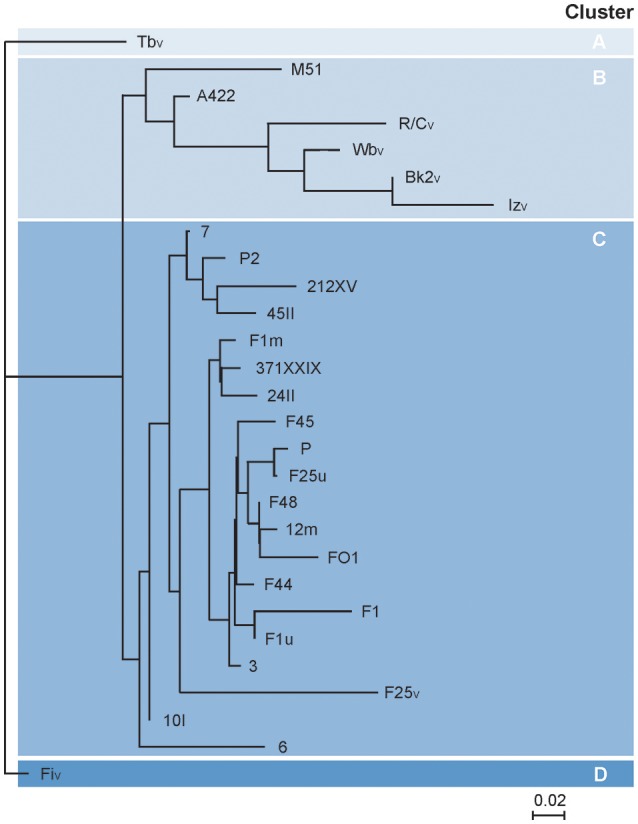
**Phylogenetic relationships of brucellaphages based on SNP analysis (Table S3)**. Analysis was conducted by using the Accelrys DS Gene Software package.

The sequence analysis of the *Brucella* reference phages disclosed several SNPs (>50 SNPS, regions A–Q, Figure 2), most of them causing amino acid exchanges or deletions. Because no data were available about the genomes of the 22 non-reference phages, we examined all SNP positions by PCR using 23 different primer pairs. The study showed that all phages differed in at least one SNP and that none of the phages exhibited the two large deletions found in Wb_V_, Bk2_V_, and R/C_V_. Taking into account all SNPs, two clusters of non-reference phages were assigned. One cluster (cluster B) consists of the phages A422 and M51 whose closest relatives are Wb_V_, Bk2_V_, Iz_V_, and R/C_V_. The remaining phages form a cluster (cluster C), which is related to cluster B (Figure 4). As the neck and tail collar protein genes of brucellaphages have been suspected to be important for host specificity (Flores et al., 2012; Farlow et al., 2014; Tevdoradze et al., 2015), we focused on the occurrence of SNPs within these genes. In the six VLA reference phages, the neck and tail collar protein genes exhibited five and seven SNPs, respectively. The analysis of the non-reference phages revealed even higher numbers of SNPs, some of which are located at the same position as in the reference phages (Table S3). A comparison of SNPs within the neck and tail collar protein genes with the host ranges of the phages did not provide evidence for amino acids that may decide on the strains that are infected. However, among 18 non-reference phages that exhibited an identical host range, 10 amino acid exchanges were observed in the neck protein and 11 in the tail collar protein, indicating that these positions do not determine specificity. Hence, even though the neck and tail collar protein genes are hotspots for nucleotide variations, it remains open which sequences are the key factor defining the host range of the phages.

## Conclusions

Lytic phages have been applied for decades to identify and discriminate *Brucella* species and biovars. Moreover, the set of reference phages used in different laboratories is basically the same. The phages were isolated many years ago and they were distributed to diagnostic laboratories worldwide. The same holds true for *Brucella* reference and type strains serving as controls. After sequencing of the first *Brucella* reference phages, it became obvious that there is not only one Tbilisi, Firenze, or Weybridge phage but some variants of the prototypes revealing distinct sequence alterations. Nucleotide variations particularly occur in genes probably involved in host specificity. Therefore, it is not surprising that the VLA reference phages investigated in this study partially exhibited a host range different from homonymous phages in other laboratories. However, it is important to point out that in our study, lytic activity was defined as the ability of the phages to form plaques, while rather unspecific lysis from without effects caused by extremely high numbers of phages were not evaluated. We think that this practice delivers more reliable data because single plaques are much easier to interpret than halos of lysis which may look very different. Results can also be biased when typing phages are propagated on different hosts, or when strains containing endogenous phages are infected. The *B. abortus* vaccine strain S19 is a well-suited host because it is susceptible to many *Brucella* phages and a Biosafety Level 2 (BSL2) organism. Thus, there are some issues that should be considered when brucellaphages are applied for typing. One main problem with lysotyping of *Brucella* strains is the similar host specificity of the reference phages. The situation could be improved by adding new phages to the typing set, which exhibit an individual host range. Our analysis of 22 non-reference brucellaphages revealed some new candidates that could be applied for routine diagnostics.

## Author contributions

JH, HS, KN, and SH designed the study. JH, CG, CJ, and JR performed the experiments. JH, CG, CJ, HS, JR, SA, and SH analyzed the data. JH, CJ, and SH wrote the manuscript and prepared the tables and figures. All authors edited the manuscript.

### Conflict of interest statement

The authors declare that the research was conducted in the absence of any commercial or financial relationships that could be construed as a potential conflict of interest.

## Supplementary material

Table S1 **Bacterial strains used in this study**.

Table S2 **Bacteriophages used in this study**.

Table S3 **Single Nucleotide Polymorphisms (SNPs) among brucellaphages**.

Table S4 **ORF analysis of brucellaphage Iz**.
