# Supplementary material for: Genetic Diversity of Brucella Reference and Non-reference Phages and Its Impact on Brucella-Typing
Source: Front Microbiol. 2017 Mar 15;8:408. doi: 10.3389/fmicb.2017.00408 (PMC5350156; doi:10.3389/fmicb.2017.00408)
Supplement: Table S1 — Bacterial strains used in this study. [file Table1.DOCX]

Supplementary Material

Genetic diversity of *Brucella* reference and non-reference phages and its impact on *Brucella*-typing

Jens A. Hammerl^*^, Cornelia Göllner, Claudia Jäckel, Holger C. Scholz, Karsten Nöckler, Jochen Reetz, Sascha Al Dahouk, and Stefan Hertwig

*** Correspondence:** Jens Andre Hammerl, Division of Epidemiology, Zoonoses and Antimicrobial Resistances, Department of Biological Safety, German Federal Institute for Risk Assessment, Diedersdorfer Weg 1, Berlin, D-12277, Germany.

[jens-andre.hammerl@bfr.bund.de](mailto:jens-andre.hammerl@bfr.bund.de)

# Supplementary Data

**Table S1. Bacterial strains used in this study**

| ***Brucella* spp. (Strain)** | **Reference** |
| --- | --- |
| ***B. abortus*** |  |
| *B. abortus* (S19) | [1] |
| *B. abortus* bv1 (544) | [1] |
| *B. abortus* bv2 (86/8/59) | [1] |
| *B. abortus* bv3 (Tulya) | [1] |
| *B. abortus* bv4 (292) | [1] |
| *B. abortus* bv5 (B3196) | [1] |
| *B. abortus* bv6 (870) | [1] |
| *B. abortus* bv7 (63/75) | [1] |
| *B. abortus* bv9 (C68) | [1] |
| ***B. melitensis*** |  |
| *B. melitensis* bv1 (16M) | [1] |
| *B. melitensis* bv2 (63/9) | [1] |
| *B. melitensis* bv3 (Ether) | [1] |
| ***B. suis*** |  |
| *B. suis* bv1 (1330) | [1] |
| *B. suis* bv2 (Thomsen) | [1] |
| *B. suis* bv3 (686) | [1] |
| *B. suis* bv4 (40) | [1] |
| *B. suis* bv5 (513) | [1] |
| ***B. ovis*** |  |
| *B. ovis* (63/290) | [1] |
| ***B. neotomae*** |  |
| *B. neotomae* (5K33) | [1] |
| ***B. canis*** |  |
| *B. canis* (RM6/66) | [1] |
| ***B. ceti*** |  |
| *B. ceti* (B1/94) | [2] |
| ***B. pinnipedialis*** |  |
| *B. pinnipedialis* (B2/94) | [2] |
| ***B. microti*** |  |
| *B. microti* (CCM 4915) | [3] |
| ***B. inopinata*** |  |
| *B. innopinata* (B01) | [4] |
| ***B. vulpis*** |  |
| *B. vulpis* (FH60HL) | [5] |
| *B. vulpis* (FH965HL) | [5] |

**References**

[1] Alton GG, Jones, L.M., Pietz, D.E. Laboratory techniques in brucellosis. Geneva: WHO no 55 Wld Hlth Org. 1975.

[2] Foster G, Osterman BS, Godfroid J, Jacques I, Cloeckaert A. *Brucella ceti* sp. nov. and *Brucella pinnipedialis* sp. nov. for *Brucella* strains with cetaceans and seals as their preferred hosts. Int J Syst Evol Microbiol. 2007;57:2688-93.

[3] Scholz HC, Hubalek Z, Sedlacek I, Vergnaud G, Tomaso H, Al Dahouk S, et al. *Brucella microti* sp. nov., isolated from the common vole *Microtus arvalis*. Int J Syst Evol Microbiol. 2008;58:375-82.

[4] Scholz HC, Nöckler K, Göllner C, Bahn P, Vergnaud G, Tomaso H, et al. *Brucella inopinata* sp. nov., isolated from a breast implant infection. Int J Syst Evol Microbiol. 2010;60:801-8.

[5] Scholz HC, Revilla-Fernandez S, Al Dahouk S, Hammerl JA, Zygmunt MS, Cloeckaert A, et al. *Brucella vulpis* sp. nov., isolated from mandibular lymph nodes of red foxes (*Vulpes vulpes*). Int J Syst Evol Microbiol. 2016;66:2090-8.
