# Supplementary material for: Genetic Diversity of Brucella Reference and Non-reference Phages and Its Impact on Brucella-Typing
Source: Front Microbiol. 2017 Mar 15;8:408. doi: 10.3389/fmicb.2017.00408 (PMC5350156; doi:10.3389/fmicb.2017.00408)
Supplement: Table S2 — Bacteriophages used in this study. [file Table2.DOCX]

Supplementary Material

Genetic diversity of *Brucella* reference and non-reference phages and its impact on *Brucella*-typing

Jens A. Hammerl^*^, Cornelia Göllner, Claudia Jäckel, Holger C. Scholz, Karsten Nöckler, Jochen Reetz, Sascha Al Dahouk, and Stefan Hertwig

*** Correspondence:** Jens Andre Hammerl, Division of Epidemiology, Zoonoses and Antimicrobial Resistances, Department of Biological Safety, German Federal Institute for Risk Assessment, Diedersdorfer Weg 1, Berlin, D-12277, Germany.

[jens-andre.hammerl@bfr.bund.de](mailto:jens-andre.hammerl@bfr.bund.de)

# Supplementary Data

**Table S2. Brucellaphages used in this study**

| **Brucella-phage** | **Phage  group** | **Propagated on** | **Origin** | **Reference** |
| --- | --- | --- | --- | --- |
| **Reference phages** | | | | |
| Tb_V_ | I | *B. abortus* S19 | VLA | This work |
| Fi_V_ | II | *B. abortus* S19 | VLA | This work |
| Bk2_V_ | III | *B. abortus* S19 | VLA | This work |
| Wb_V_ | IV | *B. abortus* S19 | VLA | This work |
| R/C_V_ | V | *B. ovis* 63/290 | VLA | This work |
| Iz_V_ | VI | *B. abortus* S19 | VLA | This work |
| **Non-reference phages** | | | | |
| A422 | n.d. | *B. abortus* S19 | VLA | [1] |
| F1 | n.d. | *B. abortus* S19 | Unknown | [2] |
| F1m | n.d. | *B. abortus* S19 | Unknown | BfR collection |
| F1u | n.d. | *B. abortus* S19 | Unknown | BfR collection |
| F25 | n.d. | *B. abortus* S19 | Unknown | BfR collection |
| F25u | n.d. | *B. abortus* S19 | Unknown | BfR collection |
| F44 | n.d. | *B. abortus* S19 | Unknown | [3-5] |
| F45 | n.d. | *B. abortus* S19 | Unknown | BfR collection |
| F48 | n.d. | *B. abortus* S19 | Unknown | [3-5] |
| FO1 | n.d. | *B. abortus* S19 | Unknown | BfR collection |
| M51 | n.d. | *B. abortus* S19 | VLA | [1] |
| P | n.d. | *B. abortus* S19 | Unknown | BfR collection |
| P2 | n.d. | *B. abortus* S19 | Unknown | BfR collection |
| 3 | n.d. | *B. abortus* S19 | Unknown | [3-5] |
| 6 | n.d. | *B. abortus* S19 | Unknown | [3-5] |
| 7 | n.d. | *B. abortus* S19 | Unknown | [3-5] |
| 10I | n.d. | *B. abortus* S19 | Unknown | [3-5] |
| 12m | n.d. | *B. abortus* S19 | Unknown | [3-5] |
| 24II | n.d. | *B. abortus* S19 | Unknown | [3-5] |
| 45III | n.d. | *B. abortus* S19 | Unknown | [3-5] |
| 212XV | n.d. | *B. abortus* S19 | Unknown | [3-5] |
| 371XXIX | n.d. | *B. abortus* S19 | Unknown | [3-5] |

VLA: OIE Brucellosis Reference Centre of the Veterinary Laboratories Agency (VLA, Addlestone, UK)

**References**

[1] Morris JA, Corbel MJ, Phillip JI. Characterization of three phages lytic for *Brucella* species. J Gen Virol. 1973;20:63-73.

[2] Hammerl JA, Al Dahouk S, Nöckler K, Göllner C, Appel B, Hertwig S. F1 and tbilisi are closely related brucellaphages exhibiting some distinct nucleotide variations which determine the host specificity. Genome Announc. 2014;2.

[3] Parnas J, Sarnecka-Szunke B. [Further characteristics of brucellaphages]. Arch Exp Veterinarmed. 1965;19:497-527.

[4] Parnas J, Sarnecka-Szunke B. [Further characteristics of *Brucella* phages. Adaptation tests, brucellicines, temperature resistance]. Z Hyg Infektionskr. 1965;151:77-80.

[5] Parnas J, Zalichta S. [Further observations on the variability of brucellaphages]. Arch Hyg Bakteriol. 1965;149:757-60.
